# The Proteome of a Healthy Human during Physical Activity under Extreme Conditions

**Published:** 2014

**Authors:** I. M. Larina, V. A. Ivanisenko, E. N. Nikolaev, A. I. Grigorev

**Affiliations:** SSC RF Institute for Biomedical Problems, Russian Academy of Sciences, Khoroshevskoye shosse, 76a, 123007, Moscow, Russia; Institute of Cytology and Genetics, Siberian Branch, Russian Academy of Sciences, Akad. Lavrentiev Ave., 10, 630090, Novosibirsk, Russia; Institute of Biochemical Physics, Russian Academy of Sciences, Kosygina Str., 4, 119334, Moscow, Russia

**Keywords:** integrative physiology, space flight, proteomics, systems biology

## Abstract

The review examines the new approaches in modern systems biology, in terms of their use for a deeper understanding of the physiological adaptation of a healthy human in extreme environments. Human physiology under extreme conditions of life, or environmental physiology, and systems biology are natural partners. The similarities and differences between the object and methods in systems biology, the OMICs (proteomics, transcriptomics, metabolomics) disciplines, and other related sciences have been studied. The latest data on environmental human physiology obtained using systems biology methods are discussed. The independent achievements of systems biology in studying the adaptation of a healthy human to physical activity, including human presence at high altitude, to the effects of hypoxia and oxidative stress have been noted. A reasonable conclusion is drawn that the application of the methods and approaches used in systems biology to study the molecular pattern of the adaptive mechanisms that develop in the human body during space flight can provide valuable fundamental knowledge and fill the picture of human metabolic pathways.

## INTRODUCTION

Proteomics (*The term was first introduced by P. James in 1997, by analogy with genomics.*) appeared in the late XXth century as a set of methods for the large-scale study of proteins [1]. Proteomics emerged as a result of a gradual development and sophistication of the classical methods used to study proteins, starting from gravimetry and photometry to disc electrophoresis, gradient, and 2D electrophoresis [2-4]. A considerable leap in the pace of development of this field of protein research took place after the possibility of using mass spectrometry to identify protein molecules was discovered. New methods of protein ionization without disturbing their primary structure appeared in the late 1980s; namely, matrix-assisted laser desorption/ionization (MALDI) and electrospray ionization (ESI) [5, 6]. The term “proteomics” recently appeared to identify the branch of systems biology that studies the protein composition of cells, tissues, body fluids, and organisms using primarily high-performance methods of mass spectrometry.

To date, tremendous progress has been made in technologies that enable one to identify proteins, measure their concentration in a sample, determine their abundance in cells, tissues and organisms, and also reveal post-translational modifications2. The number of peptides and proteins that can be identified and quantified has been steadily increasing (www.SwissProt.com).

Yet, despite the impressive achievements in implementing mass spectrometry technologies in experimental biology, the results obtained using proteomic methods have had a lesser impact on medicine than those obtained with genomic research. In our opinion, this is mainly because proteomic problems are more complicated due to the lack of methods for the amplification of protein molecules similar to PCR, and also because of the fact that the amount of proteins is much greater than that of genes (by many orders of magnitude). There are also some other complicated reasons. No doubt, such a gap between theory and practical application impedes the rate at which such discoveries as the revealing of genetic functions etc. are implemented into practical knowledge and used in clinical medicine.

## DEVELOPMENT OF PROTEOMICS: ACHIEVEMENTS AND COMPLICATIONS

The status of post-genome OMICs (*OMICs – collective name of proteomics, transcriptomics, peptidomics, metabolomics and other post-genomic disciplines.*), including proteomics, has been summarized in detail in numerous reviews [7-13]. Thus, Lander [10] noted that the decade of the post-genomic era has been characterized by intensive accumulation and cataloging of data on full sets of cellular components as a result of the intensive development of the global study of the structures of genomes, proteomes, transcriptomes, metabolomes and other “-omes.” However, the existing gap between structural success and functional implementation –understanding the functions that characterize the vital activities and the disorders in pathological conditions –devalue such discoveries. The functional analysis remains critical today for advancing towards practical application [10]. This statement has also been supported by Alberts [8].

As for proteomics, Bensimon *et al*. [7] emphasize four reasons for the lag in practical implementation (these reasons are of conceptual and technical nature). First of all, many scientists find mass spectrometry technologies to be quite complicated and requiring expensive equipment that needs constant improvement. The same can be attributed to genomics and its high-performance technologies, but the process of obtaining results using a proteomic analysis is nonlinear and it utilizes several different protocols; therefore, proteomic methods in reality appear to be objectively more complex. In summary, one can conclude that the progress achieved in proteomics is closely related to the improvement in mass spectrometry methods and increasing the accessibility to mass spectrometrybased instruments of proteomics for the wide range of scientists involved in the field of proteomics and the adjacent fields. Indeed, an analysis of the published data shows that a significant portion of high-quality results in the field of proteomics are generated by a relatively small number of laboratories. It has been noted [7] that, nowadays, from 7,000 to 10,000 human proteins can be reliably identified, and this without taking into account major proteins (present at high concentrations in samples).

When the analysis of cell line proteomes became possible, such an approach drew the attention of many researchers. However, the studies identified only approximately 100 high-abundance proteins [14-17].

Second, the studies that use high-performance methods of mass spectrometry conducted in order to identify markers show no significant advantages in the case of hypothesis-driven research, which remains the major method in life sciences. The repeating cycles of experiments with the generation and testing of hypotheses using proteomic data sets do not allow one to arrive at the expected benefit at the initial stage of the discovery of markers.

Third, it has become generally accepted that the cataloging of proteins in a sample or the predicting of their potential synthesis from a gene located on a specific chromosome (which is the main goal of the initiative of the Chromosome-Centric Human Proteome Project (C-HPP) by HUPO) are needed at the current stage but are insufficient for a biological understanding of the physical and functional interaction between proteins under conditions of dynamic molecular networks, where identifying the function of specific proteins is as important as determining the structure and function of individual proteins [2, 10, 17]. The understanding that biological processes should be studied using the dynamic networks of interacting molecules and changes in the network structure or topology determine the phenotype and underlies the new field of systems biology currently under development [7].

Fourth, the technical limitations of mass spectrometry (as the main “breakthrough” tool in proteomics) in terms of the data integrity and reproducibility of peptide identification and the protein correspondence decreases the value of the results of comparing the proteomic data obtained by various researchers and laboratories. As a result, specialized mass spectrometry teams generate large, high-quality datasets that are difficult or simply impossible to interpret and apply today, while the overwhelming majority of researchers in the field of life sciences perform analyses of small sets of proteins using methods that were developed decades ago (e.g., Western blot [18] and enzyme-linked immunosorbent assay).

It is important to mention that the number of human proteins predicted using the genomic sequence and identified experimentally increases but not at such an impressive rate as it has been predicted. For example, 11 years of HLPP (Human Liver Protein Project) studies led to the discovery of 12,168 proteins of the hepatic tissue and organelles from four main types of the hepatic tissue, and the number is expected to reach 13,000 by 2015 [19]. Currently, a total of 20,128 nucleotide sequences encoding proteins in the human genome, among which the existence of 15,646 proteins has been experimentally confirmed (75%, assuming that 1 gene = 1 protein), have been found [20, 21]. The existence of the so-called “missing proteins,” as well as the degree of uncertainty arising from the lack of a strict correspondence between the number of genes and proteins and other, already known, molecular biological principles complicate the study of the human proteome.

The proteins in the organism do not function alone. They form multiprotein complexes on the one hand and complex functional and dynamic networks on the other hand [22-25]. The organization into functional modules demonstrates the complexity and diversity of the proteome at the subcellular, cellular, and tissue levels. Understanding the organization and function of protein networks, which describe the molecular mechanisms of biological processes, is necessary to clarify the regulation (and maintenance) of the degree of health and reserves, and well as the development of human diseases (oncologic, neurodegenerative, cardiovascular ones, etc.). The study of protein interactions, including their association with non-protein molecules, and analysis of the protein networks formed by protein–protein interactions are important tools for the diagnosis, determination of disease pathogenesis, and search for molecular targets for therapeutic interventions [26, 27].

In addition, since most eukaryotic proteins are multimodular and polyfunctional [28-30], a protein acquires the ability to accomplish a range of different functions participating in various pathways. In these circumstances, eukaryotic protein networks usually interfere with each other [22, 31-33]. The tasks of evaluating the interaction between the molecular components of a biological system and the integration of such information into systems of networks and pathways that can be used to develop models, to predict the behavior of the system, constitute a serious challenge for researchers who develop bioinformatic methods of analysis for proteomics [22, 25, 33-36].

## PROTEOMICS AND SYSTEMS BIOLOGY

Proteomics and other OMICs disciplines (genomics, transcriptomics, and metabolomics) are not only new research tools and new measurement possibilities. Their emergence and development have brought new meaning to systems biology.

In their review, Edwards and Thiele [37] stated the following about the meaning of the term “systems biology”: “If then it is nothing new, why is systems biology suddenly so visible? Some have implicitly argued [38, 39] that systems biology is a mirage, no more than a rebranding of the type of holistic think - ing that some biologists and integrative physiologists have been using for decades.” As it often happens to scientific terms, the meaning of the concept “systems biology” in its current form is different from the one used previously in the aforementioned sciences. Thus, although the term “systems biology” is not new, its meaning has now changed. This is attributed to the development of technologies, especially genome sequencing, and computational and analytical platforms, such as mass spectrometry and nuclear magnetic resonance. In order to truly study a large system in its entirety, one requires the ability to fully model and measure it. Prior to the sequencing of total genomes, this had been an insurmountable experimental challenge for biologists. With the enhancement of computational power during data processing and development of genomics, transcriptomics, proteomics, metabolomics, and fluxomics1, it has become possible to study the “profiles” and models of a biological system or subsystem in its entirety. Systems biology and the so-called OMICs disciplines are not identical. Systems biology, using most of the OMICs data, goes beyond the scope of these methods [40-42]. At the same time, the term “systems biology” is understood rather narrowly by most scientists who use genomics and OMICs as a complex approach that utilizes experimental data obtained at different levels of life organization. This is mostly due to the specific understanding of the term, giving it a general meaning. A well-known physiologist, Noble *at al*. [43], in full agreement with other specialists, defines systems biology as an approach but not a field of science. Meanwhile, scientists who work in the field of systems biology consider it to be a scientific discipline that tries to study biological systems in a holistic, rather than a reductionist, way. This includes the collection of dynamic global datasets, along with phenotypic data from various levels of the biological information hierarchy, in order to identify and explain the mechanisms of the emerging characteristics (*These are the characteristics that appear in complex systems as a result of the interaction between their components; they cannot be predicted based on the characteristics of individual components. If the organization of a complex system is hierarchical, the characteristics influence each other as “top to bottom” and “bottom to top” [13, 56-59]. 2 Interactome, as the whole set of protein–protein interactions in a cell or an organism, is more complex than the proteome, and has been recently used as the measure of organism complexity [50].*) of the system [9, 25, 44].

## MATHEMATICAL MODELING AND COMPUTER TECHNOLOGIES IN MODERN BIOLOGY

One has to admit that the most fundamental difference between systems biology and OMICs disciplines and other related sciences, such as integrative physiology, is the central role of mathematical modeling and computer technologies [45-47]. It is impossible today to consider all cellular processes and simultaneously study the molecular mechanisms of a process or phenomenon even when using high-performance experimental methods. Systems biology provides the tools for solving such problems, since it incorporates the methods of mathematical and computer modeling [48]. First of all, they is the method of molecular dynamics [49], interactome mapping 2 (including experimental methods) [22], and the development of special algorithms for the construction, design, and visualization of intra- and intercellular processes and phenomena [51-53], which are used in computer modeling. It is obvious that understanding of the term “systems biology” has significantly changed, and a number of authors note that a consensus is emerging on that subject [42, 45, 54]. Given the vast amounts of data generated by the methods of genomics, transcriptomics, proteomics, and metabolomics, their manual interpretation by a researcher without the help of bioinformatics is completely ineffective or mostly impossible, and inevitably this calls for a need to apply the methods and principles of systems biology.

The genome-scale construction of the metabolic processes in a human organism with further reconstruction, published in 2007, was named Recon 1 [55]. Recon 1 provided the description of 1,496 open reading frame functions for 2,766 metabolites and 3,311 reactions located in seven intercellular compartments (cytoplasm, mitochondria, nucleus, endoplasmic reticulum, Golgi apparatus, lysosome, and peroxisome) and in the extracellular environment. This first comprehensive, genome-scale human metabolic reconstruction covers most of the known central metabolic pathways occurring in any human cell [60]. Moreover, the reconstruction made in 2007 served as a starting point for further tissue- and cell-type specific metabolic reconstructions using the data generated in OMICs studies (e. g., transcriptomic and proteomic data). Now these reconstructions are made for human macrophages [61], hepatocytes [62], myocytes, and adipocytes [63].

The major issue confronting systems biology as concerns human physiology is to fill the gaps existing in molecular networks up to their complete reconstruction.

One of the methods to pinpoint the missing reactions in the reconstructing network is to compare the results of model calculations with experimental data [64]. Numerous computational algorithms to apply in this method have been published [64-67]. Moreover, the metabolomics data of cells, tissues, and body fluids [68-70] can be used to reveal the missing links in the human metabolism. There are several different computational approaches [65, 67] that are used to find the missing candidate protein in a reaction and the corresponding genes [71, 72]. Such computational methods determine one or several reactions that take place in the organisms of other species collected in the universal protein interactions database, such as the ligand database KEGG [73], and they add them into a metabolic model, thus filling the gaps in potential missing knowledge. If experimental confirmation cannot be found in the scientific literature, one has to predict the missing genes and reactions and formulate hypotheses that require experimental testing.

## EXPERIMENTAL METHODS IN HUMAN SYSTEMS BIOLOGY

How are such methods helpful to a researcher studying extreme conditions (and what is the value in collecting data on human physiology in extreme environments employing such methods for systems biology)? The concept of disturbances plays a key role in systems biology [45]. Systems biology is based on: (1) the ability to measure all variables of interest (OMICs); (2) the presence of a conceptual framework for data interpretation (there are models); (3) and the application of the disturbance method in the experiment. Such effects on the organism, which are capable of disturbing homeostasis, allow one to define the mechanisms that help maintain a constant internal environment and preserve health resources, and the adaptive potential of an organism. However, if the object of the research is a healthy human, then the list of methods (conditions), ethically appropriate and available for exposure that lead to the decline of his homeostasis, would be quite short and would include physical activity, the use of pharmaceuticals, nutrition manipulations (e. g., the use of lipid emulsions [70] or directive changes in salt consumption [74]), functional exercise testing [75], environmental studies, including exposure to extreme temperatures, high barometric pressure and hypoxia, and, finally, space flights. Thus, extreme conditions are among the few ways to cause a decline in homeostasis in a healthy human and provide “experimental” data for systems biology. Therefore, we suggest that ecological and gravitational physiology and human systems biology are natural symbionts.

## ENVIRONMENTAL STUDIES AND SYSTEMS BIOLOGY

Only a few scientists admit the benefit of experimental data on the influence of physical exercises on a human organism for systems biology [76, 77]. At the same time, the application of systems biology methods enables one to display the whole set of proteins and metabolites inherent to the stress phase (throughout a 1.060-km non-stop cycling event) [78], and also to reveal the mechanism underlying the phenotypic response to physical activity (during the process of adaptation of fish white and red myofibrils to the training load), with the activation of metabolic networks in white myofibrils (catabolism of carbohydrates, protein synthesis, muscle contraction, and detoxification) and insufficient expression of others in red myofibrils (responsible for energy production, muscle contraction, and maintenance of homeostasis) [79]. Application of the analytical capabilities of various OMICs provided the data that helped prove the role of PGC- 1α as a transcriptional coactivator that coordinates the activation of metabolic genes (responsible for mitochondrial biogenesis) in human skeletal muscle in response to physical activity [80]; allowed one to determine the connection between genome-mediated muscle plasticity and modulation of hypoxia-specific mitochondrial biogenesis [81]; and also to establish the metabolic pathways that are activated during physical exercise, revealing several dynamically regulated miRNA–mRNA networks [82].

Systems biology studies of environmental human physiology have been started [42, 47]. Some interesting studies in the field of high-altitude genetics and proteomics have been carried out. Several studies have convincingly shown that human populations living at high altitudes experience genetic divergence. Thus, the Tibetans whose ancestors have been living at high altitudes for more than 10,000 years have acquired and inherited new mutations of the gene encoding the oxygen- sensitive hypoxia-inducible factor (HIF) [83, 84]. Studies of the proteome of the skeleton muscle biopsy samples obtained from volunteers who had spent one week at an altitude of 4,500 m [85], carried out using 2D gel electrophoresis, revealed a much larger number of proteins (involved in iron transfer and oxidative metabolism), the number of which significantly differs from that of experienced climbers after a stay at a much higher altitude. The changes in the urinary peptidome [86] and human plasma proteome [87] in response to high-altitude exposure have also been studied. In the latter case, special attention was paid to the identification of high-altitude pulmonary edema biomarkers. These and similar studies provide building blocks for coordinated efforts in systems biology and physiology in understanding the human physiological reaction to high altitudes. The extensive data on experimental hypoxia, including experiments using the methods of systems biology, have been reported in [88-95].

The ascent of a man to the highest mountain peaks initiated a surge of studies in the field of the physiological outcomes of physical activity at high altitudes. It has been established that the catabolic effects of chronic exposure to a hypoxic environment on muscles are a result of insufficient activation of hypoxiasensitive signaling pathways and suppression of the energy-intensive processes of protein translation [96]. The study of the proteome modulation caused by hypobaric hypoxia allowed one to establish that efficient use of energy-generating pathways in conjugation with an abundance of antioxidant enzymes makes the cortex less vulnerable to hypoxia than the hippocampus [97]. The experimental study of pulmonary hypertension under hypobaric hypoxia conditions showed the characteristic structural remodeling in lungs, the mechanism of which involves isoforms of heat shock protein 70 (HSP70) and protein disulfide isomerases A3 (PDIA3) [98].

The studies of the mechanisms of oxidative stress and, more broadly, cell redox homeostasis have conclusively proved the dual role of reactive oxygen species (ROS) [99]. Uncontrolled overproduction of ROS damages cellular structures, including membrane lipids, proteins, and DNA [100]. At the same time, there is increasing evidence that ROS act as secondary messengers of intracellular signaling cascades, which can cause and maintain the oncogenic phenotype of cancer cells, but they are also in - volved in senescence and apoptosis [101]. Intensive studies in this field have even led to a change in the definition of the term “oxidative stress,” making the process dependable on the changes in the real posttranslational thiol modification of proteins [102, 103]. The damage to ROS-induced signaling pathways has pathophysiological consequences that manifest themselves as disease progression (cardiovascular disorders, atherosclerosis, hypertension, ischemiareperfusion injury, diabetes, neurodegenerative diseases –Alzheimer’s and Parkinson’s, rheumatoid arthritis). A positive role of ROS is its ability to protect against infectious agents through the non-specific activation of T- and B lymphocytes, participate in the functioning of numerous signaling pathways, and induce mitogenesis [100].

## HUMAN GRAVITATIONAL PHYSIOLOGY AND SYSTEMS BIOLOGY

Finally, space flight (its influence has been studied by physiologists and physicians for more than 50 years) can be regarded as an unprecedented in the history of human evolution experience of adaptation of a healthy person to extreme environmental conditions.

Paying tribute to the pioneers of this research in the Soviet Union (L.A. Orbeli, V.V. Parin, A.V. Lebedinsky, N.M. Sisakyan, O.G. Gazenco and many others), in the USA, and also in France, Germany, and Japan, we would like to refer the reader to the fundamental monographs that analyze the long-term results in this field [104-107]. Many effects observed in astronauts after a space flight have been well described at the physiological level. In general, it appears to be a complex pattern of adaptive reactions that involve all functional systems of the body. The need for astronauts to return to Earth, to their conventional habitat, and the obligations of physicians to keep them healthy led to efforts undertaken by specialists in all, without exception, space agencies, to develop measures to impede the onset of the phase of structural adaptation to the factors the human body is exposed to in space. Nevertheless, several tissues (e.g., bone tissue) exhibit slow re-adaptation to life on Earth. Space physiology, apparently, deals with the unique pattern of adaptation of human systems, tissues, and cells, thus demonstrating its possibilities. The phenomenology of the major changes induced by the conditions of space flight includes: a negative energy balance (more energy is spent than is received) that affects various processes in a human organism [107-110], a negative water and calcium balance [111, 112] but positive sodium balance [113, 114], demineralization and modification of bone tissue structure [115], ineffective thermoregulation [116-118], changes in the biorhythms of heat production, hormone secretion activity, cardiac function [118-121], reorganization of vasomotor reaction modulation [122], endothelial dysfunction [123], muscle hypotrophy [124-126], decreased muscle tone and speed-strength properties, functional deafferentation of sensor systems that leads to impairments in movement control [127, 128], modification of lung volume, breathing biomechanics and its regulation with chemoreceptors [129, 130], and space anemia [131]. Almost every field of knowledge still has unrevealed molecular mechanisms responsible for the formation of these new stages of physiological systems.

Adaptation of the human organism to any environmental factor is performed with the help of proteins. For a long time, in accordance with analytical capabilities, working hypotheses were based on assumptions on the changes in the concentrations of working proteins or the efficiency of their performance (e.g., enzymatic activity) during adaptation. During the post-genomic era, it has become clear that such a level of study of the adaptation mechanisms will be followed by others: studies at the transcriptional level (i.e. the formation of a new set of functioning proteins) and studying the new protein complexes that are formed during adaptation, along with the protein interaction networks and new reaction cascades. These studies can be conducted with the help of systems biology, using its analytical and bio-information approaches. There are some needs that have been acknowledged by the community of gravitational physiologists but that have not been satisfied so far, such as reaching the level of OMICs. Glass [132], Jackman and Kandarian [133], Ventadour and Attaix [134] and Blottner [135] have noted that the biological effects of microgravity on the genome, proteome, transcriptome, and metabolome remain almost completely unknown.

We suggest that using the methods and approaches of systems biology to study the molecular pattern of adaptive mechanisms that is the most complicated (among all possible variants) at the present stage would both yield valuable knowledge and help to fill in the gaps in the picture of human metabolic pathways; many of these gaps have not even been considered to exist. The community of systems biologists has only begun to realize this exciting perspective. The first papers devoted to changes in the proteome of body fluids (urine and blood) in astronauts after flight [136, 137] have been published, arousing great interest, according to the number of visits on PLoSOne pages of open access (700 retrieves per week). We have studied the influence of overloads in a large-radius centrifuge [137] and breathing of the oxygen-nitrogen-argon mixture under hyperbaric conditions [138]. The characteristics of the blood and urine proteomes of a healthy human under model conditions of “dry immersion” [139] and long-term isolation [140] have been studied. Since the variability of the protein composition of human biological fluids might mask the effects of external impacts, we identified the indices of individual and group variability [141, 142]. It is necessary to take into account the parameters of group variability and the rates of manifestation of individual plasticity in order to determine functional shifts in the protein composition of body fluids during changes under conditions of the living environment, as well as disease progression. The use of direct mass spectrometric profiling of blood serum has allowed researchers to determine which proteins determine the significant group variability (CV = 42.6%) and the dependence of this parameter on age. The individual variability indices turn out to depend linearly on the length of the periods between repeated surveys, increasing from 16 to 42% for periods from 1 day to 1 year. The common changes in the blood proteome observed for space flights and model experiments were modifications of the peaks of acute phase proteins (β2- microglobulin, cystatin C) and lipid exchange (apolipoproteins CI, CIII, AII), as well as the shifts in the activity of blood proteolytic systems that can cause changes in the pattern of protein fragments.

High group and individual variability in the urinary protein profile has been noted by many scientists. We have showed that this is maintained even under the strict conditions of model experiments (with control of the intake levels of essential nutrients, fluids, level of locomotor activity, composition of the atmosphere, and sleep-wake rhythms). We observed the modification of the urinary proteome in healthy young men for 520 days with isolation in a hermoobject and managed to identify and characterize both the most plastic part of the low-molecular urinary subproteome and its constant component. Moreover, the proteins whose level in urine depended on the salt intake were discovered.

The study of the urine proteome of astronauts allowed one to identify the stable portion of the subproteome represented by 21 proteins with different tissue specificities and subcellular localizations. Three proteins (afamin, aminopeptidase A and aquaporin 2) appear in the urine of astronauts after long-term flights aboard the International Space Station; the frequency of their detection in samples is most likely related to the impact of space flight factors. The overloads experienced by astronauts at the initial and final stages of the flight can also affect the protein composition of extracellular fluid.

In the dry immersion model, the development of polyurea through a mechanism close to saluresis leads to the development of physiological proteinurea and competitively dependent sodium reabsorption in the proximal tubule of the nephron.

It is obvious that the proteins whose levels change under extreme conditions cannot be regarded as potential biomarkers of diseases, since they participate both in the natural molecular response of an organism during adaptation to a living environment and in the nonspecific component of a disease’s pathogenesis.

## CONCLUSIONS

The collaboration between physiologists and systems biologists in studying the adaptation of healthy humans to extreme environmental conditions is deepening and mutually beneficial. Researchers note that the application of systems biology methods in the field of physiological adaptation to extreme environments enables one to move away from the reductionist approaches and avoid paradoxes (e. g., the so-called “lactate paradox in hypoxia”) when interpreting data [76, 143].

*The term **hypoxia** refers to the phenomenon associated with the suppression of* *glycolysis during acclimatization to chronic hypoxia. It was shown that the* *acute phase of high-altitude adaptation is accompanied by a higher blood* *level of lactate at any period of submaximal load than under normoxia* *load, although the peak level of lactate remains unchanged. However, in* *individuals who have acclimatized to the altitude for more than 3 weeks,* *a load of the same absolute value and a maximum load cause a smaller* *increase in the blood lactate level compared to the same physical load in* *individuals in the un-acclimatized state. This phenomenon, initially regarded* *as a paradox (i.e., that does not correspond to a logical inference),* *suggested that ATP production in chronic hypoxia, apparently, does not* *depend on an increase in anaerobic glycolysis, but the production of mitochondrial* *ATP becomes better tuned to the hypoxic condition of the organism.* *Recent studies, however, have shown that the “lactate paradox” can* *only be a transitional feature of hypoxic adaptation to altitude, disappearing* *after more than 6 weeks, during the descent to the plains after a climb* *to altitudes above 5,000 m. Moreover, the decrease in the muscle ability to* *produce lactate during the period following acclimatization has not been* *shown in the studies. The question remains open as to whether the “lactate* *paradox” is caused by the decrease in lactate production in muscles due* *to the changes in the substrate preference or changes in lactate processing* *through the mitochondrial enzyme complexes MCT1 and MCT4 (monocarboxylate* *transporters 1 and 4) in muscle, or for better coupling of pyruvate* *synthesis with oxidation taking place in mitochondria. The question* *remains to be solved, along with defining a clear profile of the conditions* *under which it occurs. Several authors have suggested that a phenomenon* *analogous to the so-called “lactate paradox” can also occur in tissues other* *than muscles, in response to acute metabolic stress in chronic hypoxia.*

Now there is growing worldwide interest in collaboration between life science researchers and their colleagues (physicists, computer scientists, chemists, and mathematicians), which has been included in the agenda of the major organizations that fund science. Thus, partnerships between specialists working in the fields of systems biology/bioengineering and human physiology will become increasingly common. The new generation of scientists who are called to work in this field will become more transdisciplinary. We agree with the statement by Edwards [37] that “no longer can biology be considered a science for those who ‘cannot do maths’.” Consequently, psychologists and scientists working in life sciences should be prepared for modern challenges by expanding their knowledge in computational methods and mathematics in general to a level that will allow them to become productive systems biologists and interact with scientists from other fields. Oncoming advance of physicists and mathematicians is a more complicated process. We are not alone in this view. Paraphrasing Ideker *et al*. [45], we can say that “the contributions of crossdisciplinary scientists will be proportional to their understanding of biology.”

Thus, new approaches inherent to modern systems biology can be used for a deeper understanding of the physiological adaptation of a healthy human to extreme environments. One can certainly agree with the opinion that environmental physiology and systems biology are natural partners [144]. Studies of human adaptation to various environmental factors, as well as the study of the response of the human body to a space flight, provide a unique platform for understanding human physiology from the systems’ perspective, allowing scientists to approach homeostasis in an ethical and evolutionarily sound way. Finally, there is hope that the relationship between integrative physiology and systems biology will develop, and that the fields will be thus better understood, leading us, in turn, to a more mature and deeper understanding of a healthy person’s biology.

## Abbreviations

OMICs: biological disciplines integrated in the group of post-genomic technologies, with the names ending in -omics
MALDI: matrix-assisted laser desorption/ionization
ESI: electrospray ionization
PCR: polymerase chain reaction
HUPO: Human Proteome Organization
C-HPP: Chromosome-Centric Human Proteome Project
HLPP: Human Liver Proteome Project
KEGGDB: Kyoto Encyclopedia of Genes and Genomes database
PGC-1α: peroxisome proliferator-activated receptor-γ coactivator 1α
HIF: hypoxiainducible factor
HSP70: heat shock protein 70 kDA
PDIA3: protein disulfide isomerase family A, member 3
ROS: reactive oxygen species
CV: coefficient of variation

## References

[1] James P. (1997). Quarterly Rev.Biophys..

[2] Murray R.F., Harper H.W., Granner D.K., Mayes P.A., Rodwell V.W. (2006). Harper’s Illustrated Biochemistry. New York: Lange Medical Books/McGraw-Hill.

[3] Hey J., Posch A., Cohen A. (2008). Meth. Mol. Biol..

[4] O’Farrell P.H. (1975). J. Biol. Chem..

[5] Karas M., Bachmann D., Bahr D., Hillenkamp F. (1987). Int. J. Mass Spectrom. Ion.

[6] Fenn J.B., Mann M., Meng C.K., Wong S.F., Whitehouse C.M. (1989). Science..

[7] Bensimon A., Heck A.J.R., Aebersold R. (2012). Annu. Rev. Biochem..

[8] Alberts B. (2012). Science..

[9] Bard J. (2013). Cell..

[10] Lander E.S. (2011). Nature.

[11] Sverdlov E.D. (2010). Patol. Fiziol. Eksp. Ter..

[12] Sverdlov E.D. (2009). Biochemistry (Mosc.)..

[13] Noble D. (2012). Intersace Focus..

[14] Nagaraj N., Wisniewski J.R., Geiger T., Cox J., Kircher M., Kelso J., Pääbo S., Mann M. (2011). Mol. Syst. Biol..

[15] Beck M., Schmidt A., Malmstroem J., Claassen M., Ori A., Szymborska A., Herzog F., Rinner O., Ellenberg J., Aebersold R. (2011). Mol. Syst. Biol..

[16] Munoz J., Low T.Y., Kok Y.J., Chin A., Frese C.K., Ding V., Choo A., Heck A.J. (2011). Mol. Syst. Biol..

[17] Vidal M., Chan D.W., Gerstein M., Mann M., Omenn G.S., Tagle D., Sechi S. (2012). Clin. Proteomics..

[18] Burnette W.N. (1981). Anal. Biochem..

[19] Zhang Y., Yang C., Wang S., Chen T., Li M., Wang X., Li D., Wang K., Ma J., Wu S. (2013). Liver Int..

[20] Legrain P. (2013). Annual International Congress HUPO-2013, Yokohama, Japan. 12–18 September 2013. Book of Program & Keynote Lectures,.

[21] Radivojac P., Clark W.T., Oron T.R., Schnoes A.M., Wittkop T., Sokolov A., Graim K., Funk C., Verspoor K., Ben-Hur A. (2013). Nat. Methods..

[22] Terentiev A.A., Moldogazieva N.T., Shaitan K.V. (2009). Uspechi Biologicheskoi Chimii..

[23] Bose B. (2013). Prog. Biophys. Mol. Biol..

[24] Kitano H. (2002). Science..

[25] Naylor S., Chen J.Y. (2010). Per. Med..

[26] Brenner S. (2010). Philos Trans. R. Soc. Lond. B Biol. Sci..

[27] Brenner S., Sijnowski T.J. (2011). Science..

[28] Carbo A., Hontecillas R., Kronsteiner B., Viladomiu M., Pedragosa M., Lu P., Philipson C.W., Hoops S., Marathe M., Eubank S. (2013). PLOs Comput. Biol..

[29] Chaufan C., Joseph J. (2013). Int. J. Health Serv..

[30] Cesareni G., Gimona M., Sudol M., Yaffe M. (2004). Modular protein domains.. Weinheim, Germany: Wiley-VCH.

[31] Tong A.H., Drees B., Nardelli G., Bader G.D., Branetti B., Castagnoli L., Evangelista M., Ferracuti S., Nelson B., Paoluzi S. (2002). Science..

[32] Sommer B., Kormeier B., Demenkov P.S., Arrigo P., Hippe K., Ates Ö., Kochetov A.V., Ivanisenko V.A., Kolchanov N.A., Hofestädt R. (2013). J. Bioinform. Comput. Biol..

[33] Demenkov P.S., Ivanisenko T.V., Kolchanov N.A., Ivanisenko V.A. (2011-2013). In Silico Biol..

[34] Hood L., Heath J.R., Phelps M.E., Lin B. (2004). Science..

[35] Park S., Yang J.S., Jang S.K., Kim S. (2009). J. Proteome Res..

[36] Ivanisenko V.A., Demenkov P.S., Pintus S.S., Ivanisenko T.V., Podkolodny N.L., Ivanisenko L.N., Rozanov A.S., Bryanskaya A.V., Kostrjukova E.S., Levizkiy S.A. (2012). Dokl. Biochem.Biophys..

[37] Edwards L.M., Thiele I. (2013). Extreme Physiol. Med..

[38] Hargreaves M. (2008). J. Appl. Physiol..

[39] Greenhaff P.L., Hargreaves M. (2011). J. Physiol..

[40] Carlson R.P., Oshota O.J., Taffs R.L. (2012). Subcell. Biochem..

[41] Thiele I., Palsson B.O. (2010). Nature Protocols.

[42] Palsson B. (2008). Systems Biology. Cambridge: Cambridge Univ. Press, 2008..

[43] Kohl P., Crampin E.J., Quinn T.F., Noble D. (2010). Clin. Pharmacol. Ther..

[44] Hood L., Rowen L., Galas D.J., Aitchison J.D. (2008). Brief Funct. Genomic Proteomic..

[45] Ideker T., Galitski T., Hood L. (2001). Annu. Rev. Genomics Hum. Genet..

[46] Westerhoff H.V. (2011). Meth. Enzymol..

[47] Shublaq N., Sansom C., Coveney P.V. (2013). Chem. Biol. Drug Des..

[48] You L. (2004). Cell Biochem.Biophys..

[49] Shaitan K.V., Turley E.V., Golik D.N., Tereshkina K.V., Levtsova O.V., Fedik I.V., Shaitan A.K., Li A., Kirpichnikov M.P. (2006). Rossiiskii khimicheskii zhurnal..

[50] Stumpf M.P., Thorne T., de Silva E., Stewart R., An H.J., Lappe M., Wiuf C. (2008). PNAS..

[51] Visvanathan M., Breit M., Pfeifer B., Baumgartner C., Modre-Osprian R., Tilg B. (2007). Meth. Inf. Med..

[52] Suresh Babu C.V., Joo Song E., Yoo Y.S. (2006). Biochimie..

[53] Conzelmann H., Saez-Rodriguez J., Sauter T., Bullinger E., Allgöwer F., Gilles E.D. (2004). Syst. Biol. (Stevenage)..

[54] Wanjek C. (2011). The NIH Catalyst..

[55] Duarte N.C., Becker S.A., Jamshidi N., Thiele I., Mo M.L., Vo T.D., Srivas R., Palsson B.Ø. (2007). Proc. Natl. Acad. Sci. USA..

[56] Korn R. (2005). Biology and Philosophy..

[57] Noble D. (2013). Prog.Biophys. Mol. Biol..

[58] Noble D. (2008). Philos Trans. A Math. Phys. Eng. Sci..

[59] van Regenmortel M.H. (2004). EMBO Rep..

[60] Bordbar A., Palsson B.O. (2012). J. Intern. Med..

[61] Bordbar A., Lewis N.E., Schellenberger J., Palsson B.Ø., Jamshidi N. (2010). Mol. Syst. Biol..

[62] Gille C., Bolling C., Hoppe A., Bulik S., Hoffmann S., Hübner K., Karlstädt A., Ganeshan R., König M., Rother K. (2010). Mol. Syst. Biol..

[63] Bordbar A., Feist A.M., Usaite-Black R., Woodcock J., Palsson B.O., Famili I. (2011). BMC Syst. Biol..

[64] Qu Z., Garfinkel A., Weiss J., Nivala M. (2011). Prog. Biophys. Mol. Biol..

[65] Reed J.L., Patel T.R., Chen K.H., Joyce A.R., Applebee M.K., Herring C.D., Bui O.T., Knight E.M., Fong S.S., Palsson B.O. (2006). Proc. Natl. Acad. Sci. USA..

[66] Satish Kumar V., Dasika M.S., Maranas S.D. (2007). BMC Bioinform..

[67] Kumar V.S., Maranas C.D. (2009). PLoS Comput. Biol..

[68] Illig T., Gieger C., Zhai G., Römisch-Margl W., Wang-Sattler R., Prehn C., Altmaier E., Kastenmüller G., Kato B.S., Mewes H.W. (2010). Nat. Genet..

[69] Suhre K., Wallaschofski H., Raffler J., Friedrich N., Haring R., Michael K., Wasner C., Krebs A., Kronenberg F., Chang D. (2011). Nat. Genet..

[70] Edwards L.M., Lawler N.G., Nikolic S.B., Peters J.M., Horne J., Wilson R., Davies N.W., Sharman J.E. (2012). J. Lipid Res..

[71] Orth J.D., Palsson B.O. (2010). Biotechnology and Bioengineering.

[72] Rolfsson O., Palsson B.O., Thiele I. (2011). BMC Syst. Biol..

[73] Kanehisa M., Goto S., Hattori M., Aoki-Kinoshita K.F., Itoh M., Kawashima S., Katayama T., Araki M., Hirakawa M. (2006). Nucleic Acids Research.

[74] Jens T., Maillet A., Lang R., Gunga H.C., Johannes B., Gauquelin-Koch G., Kihm E., Larina I., Gharib C., Kirsch K.A. (2002). Am. J. Kidney Dis..

[75] Grigoriev A.I., Arzamazov G.C., Dorohova B.R., Kozjyrevskaia G.I., Morukov B.V., Natochin J.V., Noskov V.B., Chmelkov V.P. (1979). Guidelines on the use of water and saline water load samples in the assessment of the functional state of human kidneys. Ministry of Health of the Russian Federation, Moscow. USSR. 1979..

[76] VazquezA. I.O., OltvaiZ.N. I.O. (2011). PLoSOne..

[77] van Beek J.H., Supandi F., Gavai A.K., de Graaf A.A., Binsl T.W., Hettling H. (2011). Philos. Trans. A Math. Phys. Eng. Sci..

[78] Zauber H., Mosler S., von Heßberg A., Schulze W.X. (2012). Proteomics..

[79] Martin-Perez M., Fernandez-Borras J., Ibarz A., Millan-Cubillo A., Felip O., de Oliveira E., Blasco J. (2012). J. Proteome Res..

[80] Pilegaard H., Saltin B., Neufer P.D. (2003). J. Physiol..

[81] Flueck M. (2010). Exp. Physiol..

[82] Tonevitsky A.G., Maltseva D.V., Abbasi A., Samatov T.R., Sakharov D., Shkurnikov M.U., Lebedev A.E., Galatenko V.V., Grigoriev A.I., Northoff H. (2013). BMC Physiol..

[83] Beall C.M., Cavalleri G., Deng L., Elston R.C., Gao Y., Knight J., Li C., Li J.C., Liang Y., McCormack M. (2010). Proc. Natl. Acad. Sci. USA..

[84] Yi X., Liang Y., Huerta-Sanchez E., Jin X., Cuo Z.X., Pool J.E., Xu X., Jiang H., Vinckenbosch N., Korneliussen T.S. (2010). Science..

[85] Vigano A., Ripamonti M., De Palma S., Capitanio D., Vasso M., Wait R., Lundby C., Cerretelli P., Gelfi C. (2008). Proteomics..

[86] Mainini V., Gianazza E., Chinello C., Bilo G., Revera M., Giuliano A., Caldara G., Lombardi C., Piperno A., Magni F. (2012). Mol. Biosyst..

[87] Ahmad Y., Shukla D., Garg I., Sharma N.K., Saxena S., Malhotra V.K., Bhargava K. (2011). Funct. Integr. Genomics..

[88] Lai X., Nikolov S., Wolkenhauer O., Vera J. (2009). Comput. Biol. Chem..

[89] Turan N., Kalko S., Stincone A., Clarke K., Sabah A., Howlett K., Curnow S.J., Rodriguez D.A., Cascante M., O’Neill L. (2011). PLoS Comput. Biol..

[90] Stefanini M.O., Qutub A.A., Mac Gabhann F., Popel A.S. (2012). Math. Med. Biol..

[91] Baumann K., Carnicer M., Dragosits M., Graf A.B., Stadlmann J., Jouhten P., Maaheimo H., Gasser B., Albiol J., Mattanovich D. (2010). BMC Syst. Biol..

[92] Schmierer B., Novak B., Schofield C.J. (2010). BMC Syst. Biol..

[93] Mac Gabhann F., Qutub A.A., Annex B.H., Popel A.S. (2010). Wiley Interdiscip Rev. Syst. Biol. Med..

[94] Kojima T., Ueda Y., Adati N., Kitamoto A., Sato A., Huang M.C., Noor J., Sameshima H., Ikenoue T. (2010). J. Mol. Neurosci..

[95] Selivanov V.A., Votyakova T.V., Zeak J.A., Trucco M., Roca J., Cascante M. (2009). PLoS Comput. Biol..

[96] Flueck M. (2009). High Alt. Med. Biol..

[97] Sharma N.K., Sethy N.K., Bhargava K. (2013). J. Proteomics..

[98] Ohata Y., Ogata S., Nakanishi K., Kanazawa F., Uenoyama M., Hiroi S., Tominaga S., Toda T., Kawai T. (2013). Histol. Histopathol..

[99] Valko M., Leibfritz D., Moncol J., Cronin M.T., Mazur M., Telser J. (2007). Int. J. Biochem. Cell Biol..

[100] Blokhina O., Fagerstedt K.V. (2010). Plant Physiol. Biochem..

[101] Jones D.P. (2006). Rejuvenation Res..

[102] Harris C., Shuster D.Z., Roman Gomez R., Sant K.E., Reed M.S., Pohl J., Hansen J.M. (2013). Free Radic. Biol. Med..

[103] Harris C., Hansen J.M. (2012). Methods Mol. Biol..

[104] Grigorieva A.I. (2002). Orbital station “Mir”. Moscow.: LLC “Anicom”.

[105] Grigorieva A.I. (2002). Orbital station “Mir”. Moscow.: LLC “Anicom”.

[106] Grigorieva A.I. (2011). International space station. Moscow.: IBMP RAS.

[107] Leach-Huntoon C.S., Grigoriev A.I., Natochin V.Yu. (1998). Am. Astronautical Soc. Publ. SanDiego, California, USA..

[108] Stein T.P. (2013). Eur. J. Appl. Physiol..

[109] Larina I.M., Stein T.R., Leskiv M.G., Shluter M.D. (2002). Orbital station “Mir”/ Edited by Grigorieva A.I. Moscow.: LLC “Anicom”.

[110] Ajotto G.B., Himomura Y.S. (2006). J. Nutr. Sci. Vitaminol..

[111] Gazenko O.G., Grigoriev A.I., Natochin J.V. (1986). Salt and water homeostais and space flight. Moscow.: Nauka,.

[112] Morukov B.V., Noskov V.B., Larina I.M., Natochin Iu.V. (2003). Ros. Fiziol. Zh. Im. I.M. Sechenova..

[113] Gerzer R., Heer M. (2005). Curr. Pharm. Biotechnol..

[114] Drummer C., Norsk P., Heer M. (2001). Am. J. Kidney Dis..

[115] Oganov V.S., Bogomolov V.V., Bakulin A.V., Novikov V.E., Kabitskaia O.E., Murashko L.M., Morgun V.V., Kasparski R.R. (2010). Fiziol Cheloveka..

[116] Klimovitsky V.Y., Alpatov A.M., Hoban-Higgins T.M., Utekhina E.S., Fuller C.A. (2000). J. Gravit. Physiol..

[117] Lakota N.G., Larina I.M. (2002). Human Physiol..

[118] Robinson E.L., Fuller C.A. (2000). Pflügers Arch..

[119] Fuller P.M., Jones T.A., Jones S.M., Fuller C.A. (2002). Proc. Natl. Acad. Sci. USA..

[120] Larina I.M., Witson P., Smornova T.M., Chen J.M. (2000). Phisiologia cheloveka..

[121] Baevskiy R.M. (2002). Phisiologia cheloveka..

[122] FominaG.A. I.O., KotovskayaA.R. I.O., PochuevV.I. I.O., Zhernavkov A.F. (2008). HumanPhysiol..

[123] Navasiolava N.M., Dignat-George F., Sabatier F., Larina I.M., Demiot C., Fortrat J.O., Gauquelin-Koch G., Kozlovskaya I.B., Custaud M.-A. (2010). Am. J. Physiol. Heart Circ. Physiol..

[124] Ohira Y., Yoshinaga T., Nomura T., Kawano F., Ishihara A., Nonaka I., Roy R.R., Edgerton V.R. (2002). Adv. Space Res..

[125] Edgerton V.R., Zhou M.Y., Ohira Y., Klitgaard H., Jiang B., Bell G., Harris B., Saltin B., Gollnick P.D., Roy R.R. (1995). J. Appl. Physiol..

[126] Shenkman B.S., Nemirovskaya T.L., Belozerova I.N., Cheglova I.A., Kozlovskaya I.B. (1999). Report of the Acadamy of Science..

[127] Tomilovskaya E.S., Berger M., Gerstenbrand F., Kozlovskaya I.B. (2007). J. Gravit. Physiol..

[128] Kornilova L.N., Naumov I.A., Azarov K.A., Sagalovitch V.N. (2012). Aviat Space Environ. Med..

[129] Baevsky R.M., Baranov V.M., Funtova I.I., Diedrich A., Pashenko A.V., Chernikova A.G., Drescher J., Jordan J., Tank J. (2007). J. Appl. Physiol..

[130] Baranov V.M., Popova Y.A., Suvorov A.V. (2011). Biomedical research on the Russian segment
ISS.. Space biology and medicine. Moscow..

[131] Grigor’evA.I. I.O., IvanovaS.M. I.O., MorukovB.V. I.O., MaksimovG.V. I.O. (2008). Dokl. Biochem. Biophys..

[132] Glass D.J. (2003). Trends Mol. Med..

[133] Jackman R.W., Kandarian S.C. (2004). Am. Physiol. Cell Physiol..

[134] Ventadour S., Attaix D. (2006). Curr. Opin. Rheumatol..

[135] Blottner D., Serradj N., Salanova M., Touma C., Palme R., Silva M., Aerts J.M., Berckmans D., Vico L., Liu Y. (2009). J. Comp. Physiol. B..

[136] Pastushkova L.Kh., Kireev K.S., Kononikhin A.S., Tiys E.S., Popov I.A., Starodubtseva N.L., Dobrokhotov I.V., Ivanisenko V.A., Larina I.M., Kolchanov N.A. (2013). PLoS One.

[137] Kireev K.S. (2013). Kidneys and urinary tract proteins in healthy human urine proteome long-term space flight.. Author’s abstract. candidate of medical sciences. Moscow: SSC RF – IBMP RAS.

[138] Paharukova N.A., Pastushkova L.H., Popova Y.A., Larina I.M. (2010). Bulletin of Experimental Biology and Medicine..

[139] Pastushkova L.H., Dobrohotov I.V., Veselova O.M., Tiys E.S., Kononihin A.S., Novoselova A.M., Kupe M., Custo M.-A., Larina I.M. (2014). Phisiologia cheloveka..

[140] Larina I.M., Kolchanov N.A., Dobrohotov I.V., Ivanisenko V.A., Demenkov P.C., Tiys E.S., Valeeva O.A., Pastushkova L.H., Nikolaev E.N. (2012). Phisiologia cheloveka..

[141] Trifonova O., Larina I., Grigoriev A., Lisitsa A., Moshkovskii S., Archakov A. (2010). ExpertRev. Proteomics..

[142] Paharukova N.A., Pastushkova L.H., Moshkovskiy S.A., Larina I.M. (2011). Biomedicina..

[143] Murray A.J. (2009). Genome Med..

[144] Hood L.E., Omenn G.S., Moritz R.L., Aebersold R., Yamamoto K.R., Amos M., Hunter-Cevera J., Locascio L. (2012). Proteomics..

